# Efficient generation of monoclonal antibodies against peptide in the context of MHCII using magnetic enrichment

**DOI:** 10.1038/ncomms11804

**Published:** 2016-06-13

**Authors:** Justin A. Spanier, Daniel R. Frederick, Justin J. Taylor, James R. Heffernan, Dmitri I. Kotov, Tijana Martinov, Kevin C. Osum, Jenna L. Ruggiero, Blake J. Rust, Samuel J. Landry, Marc K. Jenkins, James B. McLachlan, Brian T. Fife

**Affiliations:** 1 Department of Medicine, Center for Immunology, University of Minnesota Medical School, Minneapolis, Minnesota 55455, USA; 2 Department of Microbiology and Immunology, Tulane University School of Medicine, New Orleans, Louisiana 70112, USA; 3 Department of Microbiology and Immunology, Center for Immunology, University of Minnesota Medical School, Minneapolis, Minnesota 55455, USA; 4 Department of Biochemistry, Tulane University School of Medicine, New Orleans, Louisiana 70112, USA

## Abstract

Monoclonal antibodies specific for foreign antigens, auto-antigens, allogeneic antigens and tumour neo-antigens in the context of major histocompatibility complex II (MHCII) are highly desirable as novel immunotherapeutics. However, there is no standard protocol for the efficient generation of monoclonal antibodies that recognize peptide in the context of MHCII, and only a limited number of such reagents exist. In this report, we describe an approach for the generation and screening of monoclonal antibodies specific for peptide bound to MHCII. This approach exploits the use of recombinant peptide:MHC monomers as immunogens, and subsequently relies on multimers to pre-screen and magnetically enrich the responding antigen-specific B cells before fusion and validation, thus saving significant time and reagents. Using this method, we have generated two antibodies enabling us to interrogate antigen presentation and T-cell activation. This methodology sets the standard to generate monoclonal antibodies against the peptide–MHCII complexes.

## Abstract (web summary)

Generating antibodies specific for the peptide–MHCII complexes has been challenging, with only a handful made to date. Here, the authors develop a more efficient approach to generate these antibodies, and demonstrate their potential in research and therapeutic applications.

## Graphical abstract

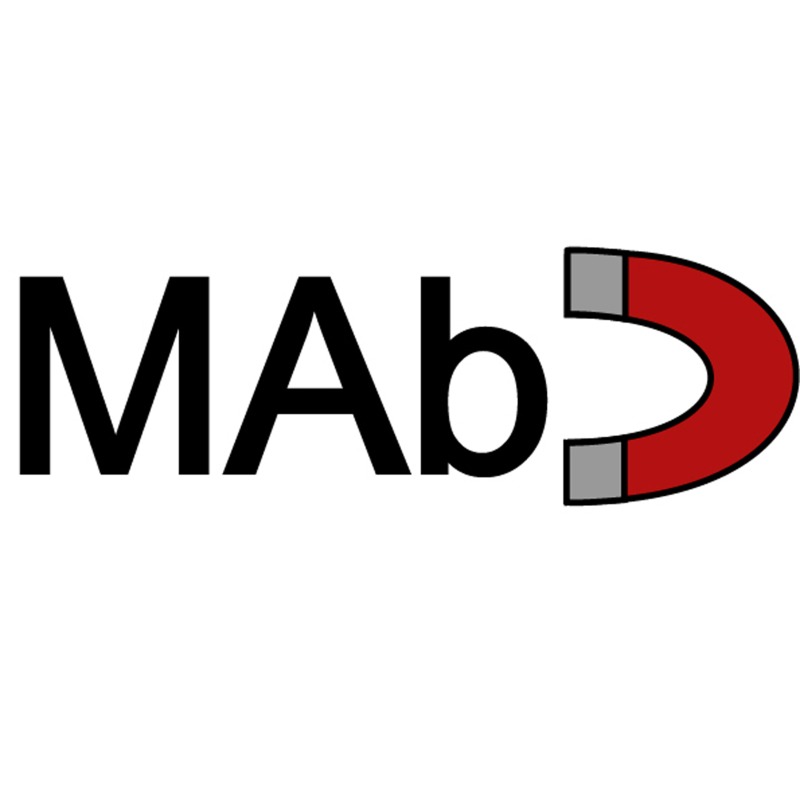

The general approach for generation of monoclonal antibodies (MAb) reactive to a defined protein antigen has been well documented since the original report in 1975 by Drs. Kohler and Milstein^1^. The utility and broad use of MAbs in biological systems earned Kohler and Milstein the Nobel Prize for medicine in 1984 (ref. 2). In this report we describe a novel methodology to specifically and reliably generate MAbs that target peptide in the context of MHCII, which has only occurred a few times since 1975 (refs 3, 4, 5, 6, 7, 8, 9).

To generate a MAb using the traditional approach, mice are immunized, the responding B cells are isolated, fused to myeloma cells with hypoxanthine–aminopterin–thymidine (HAT)-based selection, screened and sub-cloned to isolate monoclonal hybridomas^2^. Screening requires the examination of hundreds or even thousands of clones for one MAb, creating a major bottleneck. This approach typically yields <1–5% hybridomas specific for a protein target antigen causing a prominent hurdle, both in time and resources^4^. However, this method is not specifically designed to generate peptide:MHCII (p:MHCII) reactive MAbs, and B-cell tolerance against self MHC adds to the difficulty. To overcome this, we developed a novel methodology to generate MAb against a specific p:MHCII complex. B-cell clones specific for the antigen of interest are enriched immediately before myeloma fusion, thus significantly reducing the screening required. The basis for this methodology centers on having a stable p:MHCII monomeric protein linked to biotin as the B-cell antigenic target. This approach has several advantages. First, immunization with p:MHCII complexes induces a B-cell response specific for that peptide in the context of MHCII. Second, use of antigen-specific tetramers allows us to pre-screen immunized mice to confirm the expansion of p:MHCII-specific B cells. Third, it offers the ability to enrich for antigen-specific B cells^10^ while discarding B-cell clones responding to unrelated antigens. Specifically, the utility of a site-directed protein biotinylation allows for the enrichment of B cells reactive to the target protein/peptide by generating a tetrameric antigen, thus increasing the avidity of B cells for antigen and enabling the capture and enrichment of antigen-specific B cells^10^,^11^. This results in a significant time and cost saving as fewer colonies are required for screening, and a higher percentage of selected hybridomas produce MAb against p:MHCII. Finally, this enrichment approach could be used for any MAb protein target including peptides and haptens, not just p:MHCII^12^,^13^,^14^.

## Results

### Generation of p:MHCII MAb

**Figure 1:**
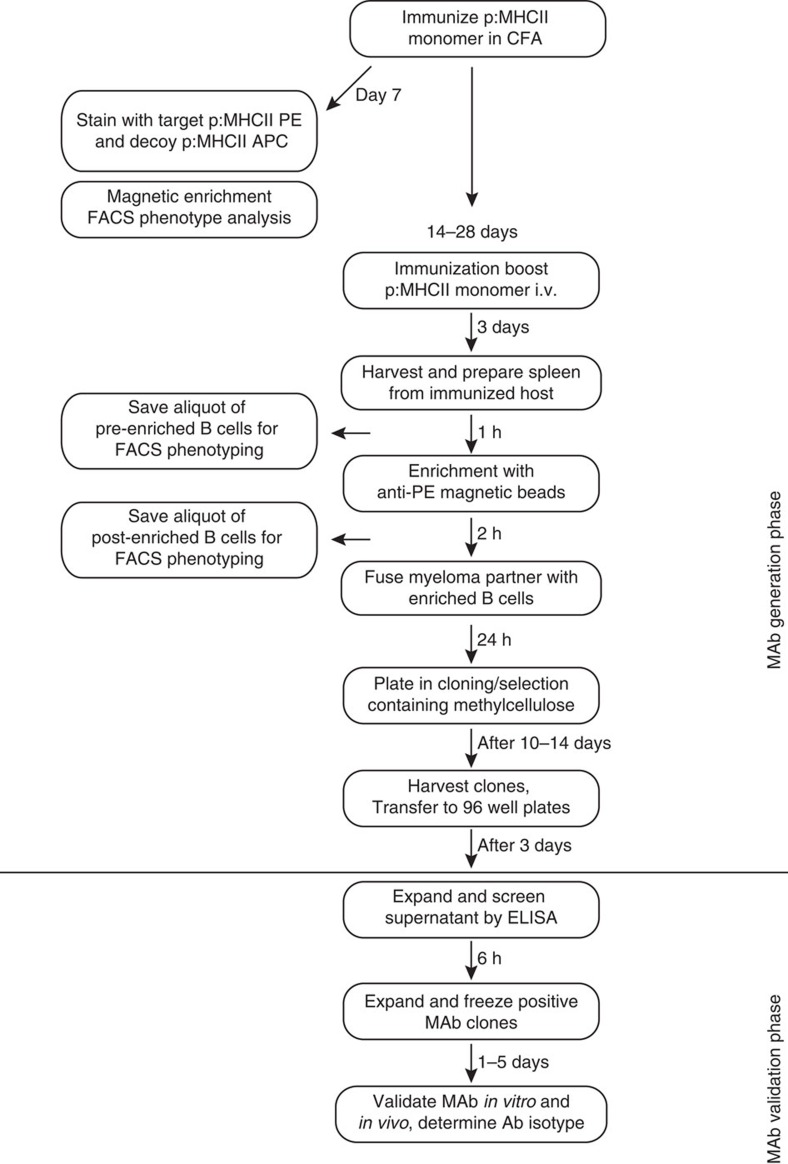
Workflow for the generation and validation of p:MHCII MAb. Mice were immunized with p:MHCII emulsified in complete Freund's adjuvant. After 7 days splenocytes from naive and immunized mice were collected and stained for specific immunogen p:MHCII-PE and decoy p:MHCII APC, streptavidin-PE-AF647, streptavidin-APC-DyLight 755, magnetically enriched using anti-PE and anti-APC magnetic beads and analyzed by flow cytometry^20^. P:MHCII-specific B cells were gated from streptavidin, APC and PE binding cells^20^. Germinal center B cells (GL7^+^ and Intracellular Ig^−^) and plasma cells (GL7^−^ and intracellular Ig^+^) were then identified from the various B-cell populations binding these distinct tetramer reagents to demonstrate successful priming. After 28 days, mice were boosted with a second immunization of p63:IA^g7^ monomeric protein intravenously. Three days following the immune boost, we preformed magnetic B-cell enrichment for splenic B cells binding to the p63:IA^g7^ tetramer:PE reagent as described in materials and methods. Enriched cells are fused with myeloma fusion partners, expanded and screened for *in vitro* and *in vivo* validation.

**Figure 2:**
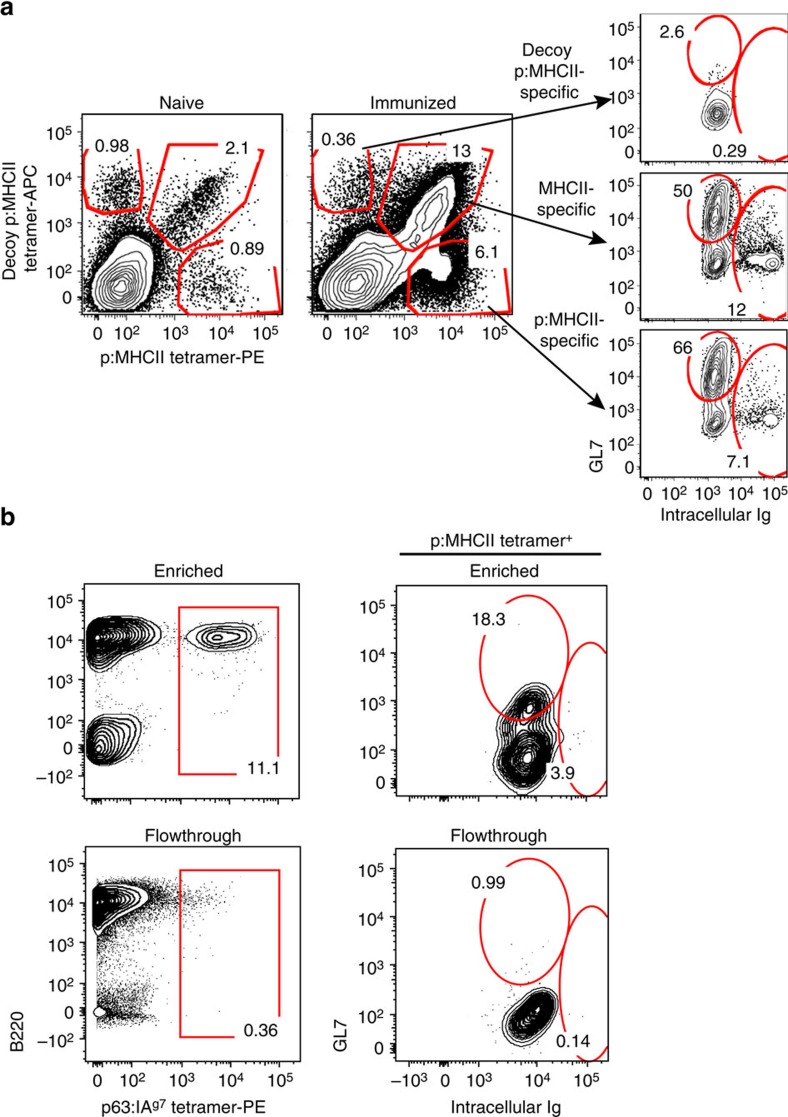
Enrichment and phenotypic analysis of p:MHCII-specific B cells. (**a**) Flow cytometric analysis of p:MHCII-specific B cells before and 7 days post immunization with p:MHCII monomer. Splenocytes from naive and immunized mice were collected and stained for specific immunogen p:MHCII-PE and decoy p:MHCII APC tetramers and magnetically enriched using anti-PE and anti-APC magnetic beads. Germinal center B cells (GL7^+^ and Intracellular Ig^−^) and plasma cells (GL7^−^ and Intracellular Ig^+^) were then identified from the various p:MHCII-specific B-cell populations binding these distinct tetramer reagents. P:MHCII-specific B cells were gated from streptavidin, APC and PE binding cells using SA-APC-DyLight 755 or SA-PE-AF647 20. (**b**) Representative flow cytometric analysis of p63:IA^g7^-enriched antigen-specific B cells obtained before myeloma fusion. Germinal center B cells (GL7^+^ and intracellular Ig^−^) and plasma cells (GL7^−^ and intracellular Ig^+^) were then identified within the p63:IA^g7^-PE tetramer specific B cells. Data are representative of two independent experiments with 2–5 mice per group.

The workflow for this methodology and the necessary steps for p:MHCII MAb generation are illustrated in Fig. 1. Generation and validation of p:MHCII MAb can be completed in <8 weeks. We were interested to develop a reagent to block T-cell receptor (TCR) recognition of a diabetes-relevant peptide^14^,^15^,^16^. We initially developed antibodies against p63 peptide in the context of IA^g7^ MHCII molecule, given that p63-activated BDC2.5 CD4^+^ T cells mediate accelerated autoimmune diabetes when transferred into wild-type non-obese diabetic (NOD) hosts^17^,^18^,^19^. We isolated splenocytes from five p:MHCII (p63:IA^g7^) immunized BALB/c mice and magnetically enriched for antigen-specific B cells using PE-conjugated p63:IA^g7^ tetramers followed by anti-PE magnetic beads^10^. To validate successful priming and expansion, we analyzed the phenotype of p:MHCII-specific B cells in naive mice compared to day 7 post immunization (Fig. 2a). Antigen-specific B cells were identified from immunized mice by p:MHCII-PE tetramer excluding those that bound to streptavidin (SA)-phycoerythrin (PE) or SA-allophycocyanin (APC) using SA-PE-AF647 or SA-APC-DyLight 755, compared to a decoy p:MHCII-APC reagent (Fig. 2a). Three distinct subsets of antigen-specific B cells (p:MHCII specific, MHCII specific and decoy p:MHCII specific)^10^,^20^ were evaluated for GL7 and intracellular Ig expression associated with mature germinal center B cells. Phenotypic analysis demonstrates the p:MHCII-PE^+^ population is enriched for mature germinal center B cells (GL7^+^ and intracellular Ig^−^) demonstrating successful priming and T-cell help (Fig. 2a). We verified the enrichment approach at day 3 post antigen boost, before hybridoma fusion. Magnetic enrichment resulted in an increase to 11.1% of the B cells staining positive for p63:IA^g7^-PE tetramer, and phenotypic markers demonstrating the presence of germinal center B cells within this population (Fig. 2b). The enriched fraction contained 2.1 × 10^7^ cells, which was 23-fold reduced compared with the starting population. These cells were subsequently fused with SP2/0 myeloma cells and plated onto ten, 100 mm plates containing semi-solid media under HAT selection. Fourteen days after plating, 190 colonies were picked and screened by enzyme-linked immunosorbent assay (ELISA). Without enrichment, we would have required 50 plates to screen 5 × 10^8^ cells. We predict that these 50 plates would have contained at least 5,000 colonies, most of which could not have been selected or screened for further analysis due to time and reagent constraints. Thus, enrichment allowed us to screen every visible colony, and saved significant time and reagents.

### Screening and *in vitro* validation of p:MHCII MAb

**Figure 3:**
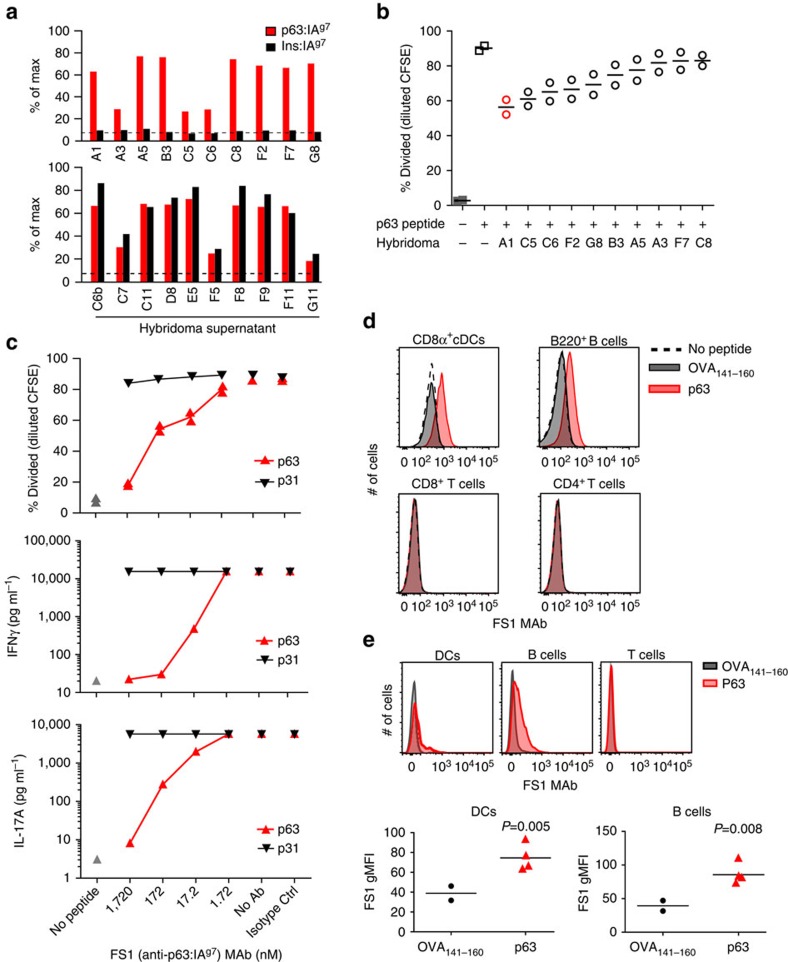
Screening and functional validation of the FS1 MAb. (**a**) ELISA results from twenty independent hybridomas presented as per cent maximum absorbance compared to anti-IA^g7^ mouse hybridoma clone 10–2.16 as positive control. Plates were coated with p63:IA^g7^ monomer and compared to InsB_9–23_:IA^g7^ monomer-coated plates. Supernatant was added and secondary antibody was used to measure binding by ELISA. Media alone was used for a negative control (dashed line). (**b**) CFSE proliferation assay in response to p63 peptide was performed using splenocytes from BDC2.5 mice. The per cent of divided CD4^+^ T cells is shown with maximum division with peptide alone (87.5%) and inhibition with each hybridoma supernatant screened. Data are representative from two independent experiments. (**c**) CFSE-labelled BDC2.5 T cells were cultured for 4 days in the presence of p63 or p31 peptide with varied concentrations of FS1 MAb or isotype control IgG1 antibody (1.72 μM). The per cent divided CD4^+^ T cells is shown for each concentration of blocking FS1 MAb compared with maximum proliferation with no antibody (no Ab) or isotype Ab. FS1 MAb effects on IFNγ cytokine production from the cultured cells (middle panel) and IL-17A cytokine production (bottom panel). Data are representative from two independent experiments in duplicate for each antigen concentration. (**d**) *In vitro* antibody staining on antigen-presenting cells following peptide pulse with p63 or OVA_141–160_ using purified clone FS1 MAb to detect p63 loaded cDCs (CD8α^+^CD11c^+^MHCII^+^, CD3e^−^, F4/80^−^), and B cells (B220^+^,MHCII^+^,CD11c^−^,CD3ɛ^−^,F4/80^−^) but not CD4^+^ or CD8^+^ T cells (CD3ɛ^+^, CD11c^−^, CD11b^−^, B220^−^, F4/80^−^) compared with no p63 peptide negative control. Data are representative from three independent experiments. (**e**) *In vivo* staining of antigen-presenting cells with FS1 MAb following footpad immunization. NOD mice received p63 or OVA_141–160_ peptide and 1.5 h following injection the popliteal lymph node was collected and stained for antigen-specific presentation using biotinylated FS1 MAb. FS1 MAb staining was detected on DCs and B cells, but not T cells using fluorochrome-linked streptavidin. Statistical significance was calculated using a two-tailed Student's *t*-test. Data are representative from two independent experiments with 2–4 mice per group.

After expansion of each colony, secreted antibody in the culture supernatant was assessed for binding to p63:IA^g7^ compared to decoy InsB_10–23:_IA^g7^ by ELISA. Thirty-two of the 190 colonies produced antibodies that bound to both, p63:IA^g7^ and InsB_10–23_:IA^g7^, indicating specificity for an IA^g7^ epitope (Table 1). In contrast, 11 hybridomas produced antibodies that bound only p63:IA^g7^ (34.4% success rate for p:MHC or 5.8% overall), suggesting the desired specificity for this peptide bound to IA^g7^ (Table 1). Figure 3a illustrates 20 clones, 10 that reacted to both p63:IA^g7^ and InsB_10–23_:IA^g7^ (bottom), and 10 that are unique for p63:IA^g7^ (top). We further characterized these 10 clones that uniquely bound p63:IA^g7^ for TCR blocking ability to limit *in vitro* antigen-specific T-cell proliferation (Fig. 3b). Splenocytes were isolated from TCR transgenic BDC2.5 mice, labelled with carboxyfluorescin succinimidyl ester (CFSE) and cultured with p63 peptide in the presence or absence of hybridoma supernatant for 4 days. BDC2.5 splenocytes incubated with peptide alone resulted in 87.5% CD4^+^ T cells proliferating, while T cells incubated with peptide plus hybridoma A1 limited BDC2.5 T-cell proliferation to 56% (Fig. 3b). The remaining nine hybridomas screened had varying degrees of inhibition (Fig. 3b). We then used an isotype-specific ELISA to determine that A1 antibody was IgG1. A large-scale purification was next performed to obtain purified MAb from hybridoma A1 (named FS1). Using the FS1 MAb (anti-p63:IA^g7^) we performed an *in vitro* dose–response assay and demonstrated 80.5% specific reduction in proliferation with 1.72 μM FS1 MAb (Fig. 3c). In contrast, the FS1 MAb only reduced BDC2.5 T-cell proliferation to another BDC2.5 mimetope (p31) by 5.6% compared with an isotype control (Fig. 3c). p63-activated BDC2.5 T cells demonstrated 99.85% reduction in IFNγ production when cultured with 1.72 μM of FS1 MAb, compared with an isotype control (Fig. 3c). Importantly, IFNγ production by p31-activated BDC2.5 T cells was not altered (Fig. 3c). We noted a similar trend with IL-17A (Fig. 3c). Taken together, these findings illustrate the specificity of the FS1 MAb as p31 differs from p63 by two amino acids at positions P-1 and P1 of the MHCII binding pocket^21^. As an extension of specific binding, splenocytes from NOD mice were p63 peptide-pulsed and stained with labelled FS1 MAb illustrating CD8α^+^ cDCs (dendritic cells) and B220^+^ B cells stained positive for p63 peptide but not ovalbumin peptide (OVA_141–160_) control (Fig. 3d). The uniform histogram shift suggests a large portion of the DCs and B cells stained with varying levels of FS1 MAb demonstrating peptide presentation *in vitro* (Fig. 3d). Importantly, CD4^+^ and CD8^+^ T cells did not stain positive for the FS1 MAb (Fig. 3d). Immunostaining was next performed to demonstrate peptide binding to MHCII *in vivo*. NOD mice were injected with p63 or OVA peptide in the footpad and 1.5 h later popliteal lymph node cells were stained with FS1 antibody to identify p63-loaded antigen-presenting cells (Fig. 3e). Both DCs and B cells had significantly increased FS1 MAb staining in response to p63 peptide-pulsed compared to OVA peptide (*P*=0.005 and *P*=0.008, respectively), while T cells showed no specific staining (Fig. 3e).

**Figure 4:**
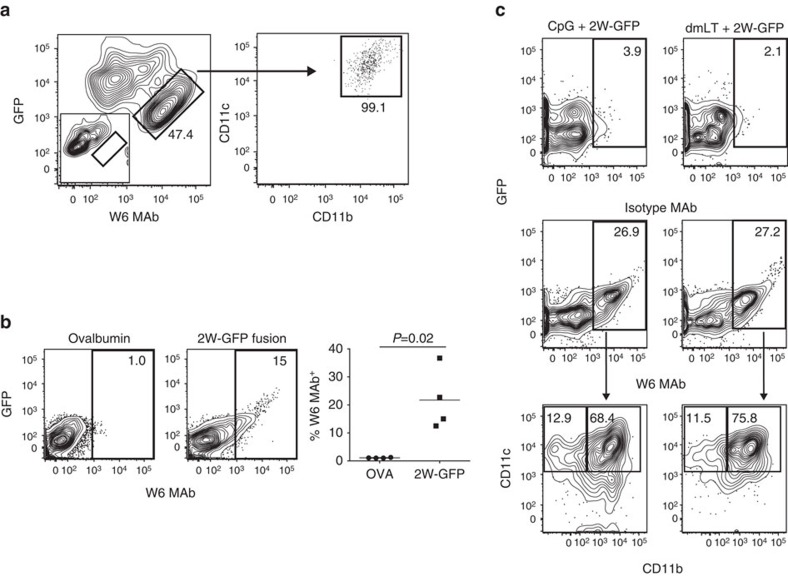
Functional validation of W6 (2W:IA^b^) MAbs. (**a**) *In vitro* antigen presentation on bone marrow-derived dendritic cells isolated from C57BL/6 mice. Cells were stained with W6 MAb after 24 h pulse with GFP covalently linked to 2W peptide. GFP and W6 MAb double-positive cells are shown compared to isotype control (insert). Data are representative from two independent experiments with 3 mice per group. (**b**) *In vivo* immunostaining of 2W antigen-presenting cells. Mice were immunized intradermally in the ear with either Ovalbumin (OVA) or 2W-GFP. 24 h post immunization, cervical lymph nodes were collected, dissociated and gated for lymphocyte size, singlets, CD19^−^ and MHCII^+^. Statistical significance was calculated using a two-tailed Student's *t*-test. Data are representative of two independent experiments with four animals per group. (**c**) Mice were immunized with 2W-GFP and either CpG or dmLT^23^. Twenty-four hours later, draining lymph nodes were assayed using W6 MAb. Shown are percentages of CD19^−^, CD11b^+^ CD11c^+^ dendritic cells. Data are representative from four independent experiments with 2–3 mice per group.

Using this novel methodology, we also generated an antibody specific for the peptide 2W^12^,^22^ bound to IA^b^ (named W6). Using this reagent, we validated *in vitro* antigen loading and presentation using bone marrow-derived dendritic cells that were pulsed with green fluorescent protein (GFP)-linked 2W peptide. Results in Figure 4a demonstrate, 47% of the GFP-positive cells were W6 MAb (anti-2W:IA^b^) positive and were mostly CD11c^+^CD11b^+^ double-positive cells. We next validated *in vivo* antigen loading and presentation. C57BL/6 mice were immunized intradermally with either ovalbumin protein or 2W-GFP. At 24 h post injection, MHCII^+^ antigen-presenting cells from the draining lymph node had increased W6 MAb reactive populations (15%) compared with 1% of controls (Fig. 4b). In a separate *in vivo* model, we used the W6 MAb to identify antigen-presenting cells immunized with 2W peptide and two different adjuvants. C57BL/6 mice were immunized with 2W-GFP and either 5′-cytosine-phosphate-guanine-3′ (CpG) or double-mutant heat-labile toxin (dmLT)^23^. Twenty-four hours later, draining lymph nodes were assayed by flow cytometry for antigen-specific presentation using the W6 antibody. Shown in Fig. 4c, the W6 MAb identified 27% of the DCs containing GFP compared with 3% in the isotype control group and ∼70% these cells were CD11b^+^CD11c^+^CD19^−^ dendritic cells. These results demonstrate that W6 MAb can identify different subsets of antigen-presenting cells *in vivo*.

### *In vivo* functional validation of p:MHCII MAb

**Figure 5:**
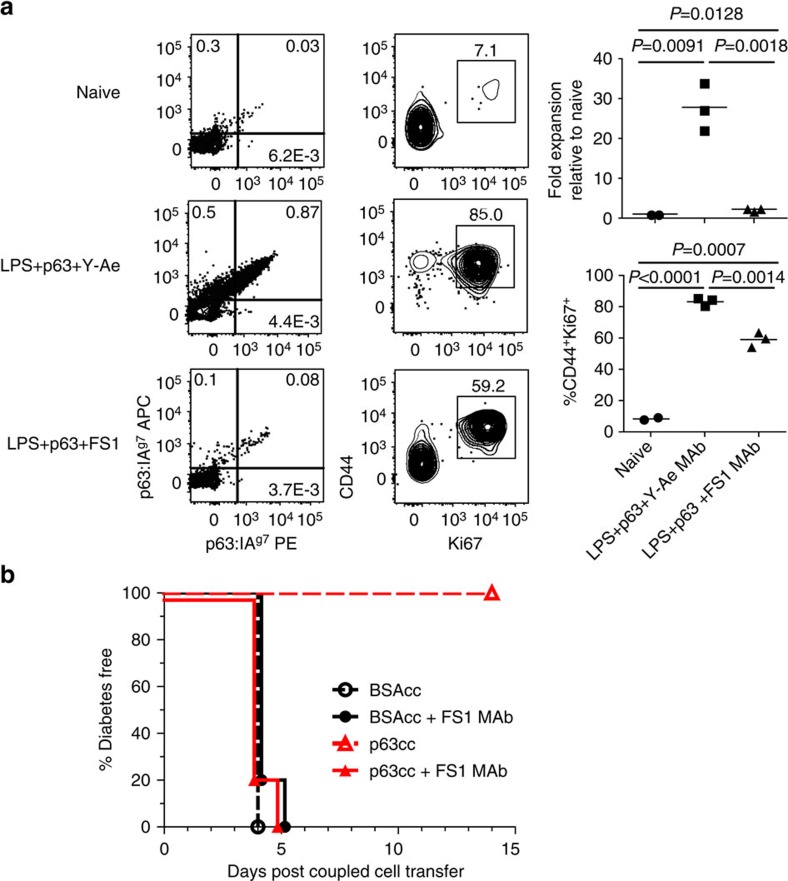
*In vivo* blockade of T-cell proliferation and prevention of T-cell tolerance following FS1 administration. (**a**) *In vivo* blockade of antigen-specific proliferation and cell cycle progression 4 days after i.v. administration of FS1 MAb plus p63 and LPS compared to Y-Ae (anti-IEα:IA^b^), control treatment or untreated naïve mice. P63:IA^g7^ tetramer PE and APC double postive cells from the spleen were enriched and gated on lymphocyte size, singlets, live cells, B220^−^, CD11c^−^, CD11b^−^, CD3e^+^ and CD4^+^. Statistical significance was calculated using a two-tailed Student's *t*-test. Data are representative from two independent experiments with 2–3 mice per group. (**b**) *In vivo* blockade of antigen-specific tolerance using FS1 MAb to prevent ethylene-carbodiimide antigen-fixed-coupled cell tolerance. NOD mice received activated BDC2.5 TCR transgenic CD4^+^ T cells to induce diabetes. Ten mice per group received either p63-coupled cells or BSA-coupled cells. Five mice per treatment received FS1 MAb or control. Mice were followed daily for diabetes after day 3 by blood glucose measurements. Data are representative from two independent experiments.

Next, we sought to determine whether MAbs directed against p:MHCII could prevent TCR recognition *in vivo* to limit T-cell proliferation. NOD mice were challenged with p63 peptide plus lipopolysaccharide (LPS) with FS1 MAb or Y-Ae^3^ (anti-Eα:IA^b^) as a negative control. Four days post challenge we measured a significant reduction in antigen-specific T-cell expansion with FS1 MAb administration (Fig. 5a). Using dual fluorochrome tetramer staining and flow cytometry, we detected 30-fold expansion of p63 specific T cells stimulated with p63+LPS+Y-Ae MAb control, compared with only a twofold expansion with p63+LPS+FS1 MAb over naive mice (Fig. 5a). In addition to decreased expansion, we also determined that the FS1 MAb decreased activation and cell cycle progression (Fig. 5a). Next, we used the FS1 MAb *in vivo* to prevent antigen-specific tolerance, resulting in rapid autoimmune diabetes^19^,^24^. Specifically, we transferred activated BDC2.5 T cells after 4 days of *in vitro* stimulation into wild-type NOD pre-diabetic recipients followed by injection of ethylene-carbodiimide (ECDI)-fixed p63 peptide-coupled cells (p63cc) to induce tolerance^14^,^16^ and either control or FS1 MAb and monitored the mice for diabetes. P63cc completely prevented diabetes induction in 100% of the mice, while control bovine serum albumin-coupled cell-treated mice developed severe diabetes (Fig. 5b). Mice given p63cc and FS1 MAb developed diabetes, indicating the FS1 MAb prevented the induction of antigen-specific tolerance *in vivo*.

**Figure 6:**
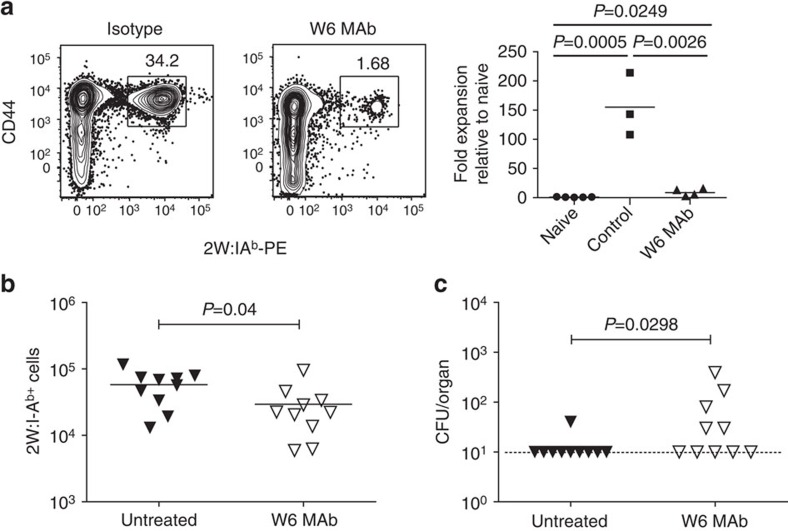
*In vivo* blockade of T-cell proliferation and prevention of bacterial clearance following W6 administration. (**a**) C57BL/6 mice were infected with *Listeria monocytogenes* expressing 2W. Seven days post infection and W6 antibody treatment, 2W-specific cells were magnetically enriched from the spleen. Cells were gated on lymphocyte size, singlets, CD19^−^, F4/80^−^, CD11c^−^, CD11b^−^, CD3e^+^ and CD4^+^. Shown are representative FACS plots for CD44^hi^ versus 2W:IA^b^ tetramer PE^+^ cells and fold expansion of antigen-specific cells relative to uninfected mice. Data are representative of two independent experiments with 3–5 mice per group. (**b**,**c**) Wild type 129S1 mice were infected with *Salmonella Typhimurium* expressing 2W, and 14 days following infection mice were treated with blocking W6 MAb. At day 35 post infection (21 days following MAb administration), (**b**), the number of 2W-specific T cells was determined from the spleen as described in **a** and (**c**) the colony forming units for the spleen was determined. Statistical significance was calculated using a two-tailed Student's *t*-test. Data include 10 mice from two independent experiments.

The W6 MAb was next evaluated for its ability to block 2W-specific *in vivo* expansion of antigen-specific T cells in response to an acute or chronic bacterial infection. C57BL/6 mice were administered W6 MAb and infected with *Listeria monocytogenes* expressing 2W^25^. Seven days later the number of activated 2W-specific cells was decreased by 146-fold in response to W6 MAb compared with no antibody control (Fig. 6a). To test *in vivo* 2W-specific CD4^+^ T-cell responses and their contribution to bacterial clearance in a chronic infection, wild type 129S1 mice were infected with 10^8^ *Salmonella Typhimurium* expressing 2W peptide^26^,^27^. The mice received a single dose of W6 blocking antibody 14 days post infection and were sacrificed at day 35 to evaluate antigen-specific T-cell proliferation and colony forming units. Infected mice treated with the W6 MAb had significantly lower 2W-specific CD4^+^ T cells, higher bacterial burden, and did not clear the *Salmonella* infection (Fig. 6b,c). These data highlight the critical importance of a single antigen-specific T-cell population and the blocking ability of the W6 MAb to prevent *in vivo* pathogen clearance.

To compare the affinity of the FS1 and W6 MAb with previously published reagents, we performed a direct side-by-side comparison with known IA^g7^ or IA^b^ specific antibodies. The results are shown in Table 2, and illustrate that the FS1 MAb (anti-p63:IA^g7^) has 100-fold higher affinity (1.7 × 10^−11^) than the 10–2.16 MAb^28^ (anti-IA^g7^) (2.9 × 10^−9^ *K*_D_ (M)). The W6 MAb (anti-2W:IA^b^) had an affinity comparable to known IA^b^ antibodies (Y3P^29^, Y-Ae^3^ and AF6-120.1 (ref. 30)). These results suggest that the two MAb generated had comparable or higher affinity than conventional approaches used to develop MAb.

## Discussion

Using this methodology we have generated hybridomas producing six novel anti-peptide:MHCII MAb, and for two of these presented here, we demonstrate the high affinity for antigen and biological capacity to limit TCR engagement, prevent subsequent T-cell activation, label antigen-presenting cells, and *in vivo* use to prevent tolerance induction and bacterial pathogen clearance. We have demonstrated that this novel methodology is highly efficient due to pre-screening and enrichment, saving both time and resources (Table 1). This approach will be useful to generate additional p:MHCII-targeted MAbs as very few exist. The most well-known is the Y-Ae antibody (anti-Eα:IA^b^), which recognizes Eα_52–68_ bound to IA^b^ MHCII molecules, and was used to understand central tolerance and alloreactive antigen presentation^3^,^31^,^32^. The efficiency of Y-Ae generation has not been described in the literature^3^, however, two more recent reports address this issue. The generation of MAbs specific for two different peptides of hen egg lysozyme (HEL), specifically two HEL_46–61_:IA^k^ (clones B6G and C4H) MAb were identified by screening 500 clones^8^. An additional clone specifically recognizing HEL_116–129:_IA^k^ (D8H) was identified by screening 500 different colonies. However, these clones were found to also weakly stain cells expressing IA^k^ in a HEL-independent manner^8^. In a separate report, Dadaglio *et al.*^9^ generated a clone specific for HEL_48–62_:IA^k^ (Aw3.18) by screening 1,000 colonies. Thus, the efficiency of generating a MAb clone using conventional approaches has been reported to range from 1:250 to 1:1,000 or (0.1–0.4%). In Table 1, we report a range of 1:18–1:115 using the novel methodology described here (0.9–13.5%). Taken together, our success rate is 2.25 to 33.75-fold higher than traditional approaches resulting in a higher efficiency. The fact that fewer clones are required for positive identification also results in a significant advantage of lower costs and fewer hours required for hybridoma screening.

More recently, an antibody against insulin B peptide_9–23_ in IA^g7^ (anti-InsB_9–23_:IA^g7^) was generated and shown to delay diabetes in NOD mice^4^. In the current study, the FS1 MAb was generated against a diabetes-relevant peptide in the context of IA^g7^. Here, we demonstrate successful blockade of antigen-specific tolerance using FS1 MAb causing rapid diabetes (Fig. 5b). Future work using FS1 MAb will characterize the role for polyclonal or monoclonal T cells during spontaneous autoimmune diabetes and tolerance^16^,^33^. The W6 reagent was generated to understand T-cell responses during both homoeostasis and bacterial pathogenesis, as the 2W peptide can be engineered into a pathogen of interest. Future work with this reagent will characterize cells presenting antigen in response to bacterial infections or immunization with different adjuvants.

One limitation to this approach centers on possessing knowledge of the antigen target, availability of p:MHC monomers for immunization and tetramers for B-cell enrichment. With advances of our understanding of peptide binding to both MHCI and MHCII, enhanced algorithms can be developed to better predict peptide register binding leading to greater numbers of p:MHC monomers^6^,^34^,^35^. This, combined with high throughput generation of p:MHCII monomers by peptide exchange^36^ would alleviate the current limitations of this approach. The generation of MAb targeting mouse p:MHC class I (MHCI) could also be performed using this methodology. In addition, human MAb targets against peptides in HLA class I or II would also be amenable to this approach, thus offering great potential for human immunotherapy in the context of autoimmunity, tumour immunity or allograft rejection. The ability to specifically block antigen presentation to T or B cells during bacterial or viral pathogenesis could provide mechanistic insight for immunity and regulation, particularly with respect to persistent infection. Finally, the use of MAb specific for p:MHC allows the study of specific subsets of antigen-presenting cells during every aspect of immune recognition ranging from immune homoeostasis to defining novel roles for multiple subsets of antigen-presenting cells responding to vaccination and infection.

We anticipate the need for immunotherapeutics in the form of MAbs directed against p:MHCII will increase as new pathways and novel antigens are identified during disease. These targets could include not only foreign antigens, but also allogeneic antigens during transplantation, tumour neo-antigens, self-proteins targeted during autoimmunity, or bacterial and viral antigens from infected cells. As these targets are identified, there will be a great need to limit antigen-specific T-cell responses, and the methodology described here offers a more efficient approach over conventional protocols.

## Methods

### Mice

Female NOD mice (6–8 weeks of age) were purchased from Taconic. Female C57BL/6 (6–8 weeks of age), female 129S1 (6–8 weeks of age) and female BALB/c mice (8–10 weeks of age) were purchased from the Jackson Laboratory. Female (6-week-old) NOD.BDC2.5 Thy1.1 transgenic mice were bred under specific pathogen-free, barrier facility at the University of Minnesota. Animals were housed under specific pathogen-free, barrier facility in accordance with NIH guidelines. All animal procedures were approved by the University of Minnesota or Tulane Institutional Animal Care and Use Committee.

### Peptides

Peptides used for *in vivo* immunization and *in vitro* stimulation, and peptide pulsing include p63 (RTRPLWVRME), p31 (YVRPLWVRME), OVA_141–160_ (CARELINSWVESQTNGIIRN) (Genemed Synthesis), 2W (EAWGALANWAVDSA) (Genscript).

### Peptide:MHCII monomers and tetramers

p63:IA^g7^ (RTRPLWVRME), InsB_10–23_:IA^g7^ (HLVERLYLVCGEEG), 2W:IA^b^ (EAWGALANWAVDSA) and LLO:IA^b^ (NEKYAQAYPNVS) was either from the NIH tetramer core facility (Emory University) or produced using the S2 insect-cell expression system^12^,^13^,^14^. Briefly, peptide:IA^g7^ or peptide:IA^b^ molecules were expressed in *Drosophila* S2 cells using the DES *Drosophila* Expression System kit (Invitrogen). The S2 cells were co-transfected using calcium phosphate, with plasmids encoding the alpha chain, the peptide-linked beta chain, BirA ligase and a blasticidin resistance gene at a molar ratio of 9:9:9:1 for IA^b^ and IA^g7^. Transfected cells were selected in blasticidin-containing Schneider's *Drosophila* medium (Invitrogen) with 10% fetal bovine serum (FBS), 100 U ml^−1^ penicillin/streptomycin (Gibco) and 20 μg ml^−1^ gentamycin (Invitrogen) for 1 week at 28 °C, passaged into serum-free media containing 25 μg ml^−1^ blasticidin (Invivogen), and scaled to 0.5 l cultures in 2 l shaker flasks maintained at 150 r.p.m. When cell densities reached 5 × 10^6 ^ml^−1^, monomer expression was induced by the addition of 0.8 mM copper sulphate. Peptide:IA^b^ or IA^g7^ heterodimers were purified from supernatants 8 days later by immobilized metal ion affinity chromatography using a His-Bind purification kit (Novagen-EMD Biosciences) and eluted using 1M imidazole. The biotinylated pMHCII heterodimers in the eluate were then affinity purified using Monomeric Avidin UltraLink (Pierce). Bound peptide:IA^b^ or IA^g7^ molecules were eluted with 2 mM biotin in PBS and excess-free biotin was removed by centrifugation and four washes with 12 ml PBS using a 30 KD cut-off Amicon Ultra-15 filter (Millipore). Tetramers were produced by incubating p:MHCII monomers with streptavidin-APC (Prozyme # PJ27S) or streptavidin-PE (Prozyme #PJRS27) at a 4:1 molar ratio. For the detection of SA and fluorochrome specific B cells, AF647 was conjugated to SA-PE (Prozyme) for 60 min at room temperature using an antibody labelling kit (ThermoFisher Scientific) and free AF647 was removed by centrifugation in a 30 KD molecular weight cut-off filter. The concentration was then adjusted to 1 μM PE based on the absorbance at 565 nm using a nandrop spectrophotometer (ThermoFisher Scientific). Similarly, SA-APC (Prozyme) was conjugated to DyLight 755 using an antibody labelting kit (ThermoFisher Scientific) and the concentration was adjusted to 1 μM APC based on the absorbance at 650 nm.

### Antigen-specific B-cell enrichment and phenotyping

BALB/c mice were immunized with 50 μg of pMHCII emulsified in complete Freund's adjuvant (CFA, Sigma) subcutaneously in the base of the tail. Seven days post immunization single-cell suspensions from spleen and pooled lymph nodes (inguinal, brachial, cervical and axillary) were prepared by forcing the tissue through a 100 μm cell strainer using the plunger end of a 1 ml syringe, washed with RPMI containing 2% FBS and resuspended in 100 μl Fc block (2.4G2, 0.05% sodium azide). The cells were next incubated with 5 nM SA-PE-AF647, and SA-APC-DyLight755 for 10 min at 25 °C, followed by peptide:MHCII-conjugated PE and APC tetramers at 10 nM for 25 min on ice in a final staining volume of 200 μl. The cells were then washed with 12 ml PBS+2% FBS, resuspended in 150 μl of PBS+2% FBS, mixed with 25 μl anti-PE and anti-APC MicroBeads (Miltenyi Biotec) and incubated for 25 min at 4 °C. The cells were then washed with 12 ml PBS+2% FBS, resuspended in 3 ml PBS+2% FBS and applied to a magnetized LS column (Miltenyi Biotec). The column was washed three times with 3 ml PBS+2% FBS, removed from the magnet and the cells were eluted in 5 ml PBS+2% FBS. All the eluted cells and 1/20th of the flowthrough were then centrifuged and stained with surface antibodies IgM-BV421 (RMM-1, BioLegend), B220-PE-Cy7 (RA3-6B2, Tonbo Biosciences), CD38-AF700 (90, eBioscience), GL7-FITC (GL7, eBioscience), IgD-BV786 (11-26 c.2a, BD Biosciences), CD11b-BV510 (M1/70, BD Biosciences), CD11c-BV510 (N418, BioLegend), F4/80-BV510 (BM8, BioLegend), CD90.2-BV510 (53-2.1, BioLegend), Live/Dead Ghost 510 dye (Tonbo Biosciences) for 30 min at 4 °C. Next, the cells were fixed with Cytofix/Cytoperm solution (BD Biosciences) for 20 min at 4 °C, washed twice with permeabilization buffer and stained with intracellular antibodies IgG (H+L)-AF350 (polyclonal, ThermoFisher Scientific) for 30 min at 4 °C in permabilization buffer before the flow cytometry analysis on a LSRII Fortessa instrument (Becton Dickinson) equipped with five laser lines (355, 405, 488, 561 and 640 nm). All antibodies were used at a 1:100 dilution for staining, except CD90.2, which was diluted 1:500. The data were analyzed using FlowJo software (v.10).

### Isolation and fusion of antibody-producing B cells

BALB/c mice were immunized subcutaneously in the base of the tail with 50 μg of p:MHCII monomer emulsified in complete Freunds' adjuvant. Twenty eight days later each mouse was boosted by intravenous (i.v.) injection of 100 μl total volume containing 25 μg of p:MHCII in PBS. Three days after boost a single cell suspension was made from pooled spleens and draining lymph nodes. Cells were stained in 150 μl of complete media (DMEM, 10% FCS, β-ME, pen/strep, nonessential amino acids) containing 13.3 nM PE-conjugated p:MHCII/mouse and incubated on ice for 25 min. Cells were washed with complete media and resuspended in 200 μl of complete media containing 50 μl of anti-PE microbeads (Miltenyi Biotec)/mouse and incubated on ice for 25 min. Cells were washed with complete media and resuspended in 5 ml of complete media/spleen. The cell suspension was then applied to a pre-equilibrated LS magnetic column (Miltenyi Biotec) and washed 2 × with 3 ml of complete media. The cells were eluted from the column in 5 ml medium A (Stem Cell Technologies), centrifuged and enumerated. The enriched B cells were fused with SP2/0 mouse myeloma cells using the HY Hybridoma Cloning Kit according the manufacturer's protocol using method A (Stem Cell Technologies). A small portion of the enriched cells and flowthrough was stained as described above to determine antigen-specific B-cell purity and phenotype before hybridoma fusion.

### Hybridoma selection and specificity screening

Twelve days post-HAT selection, individual colonies were handpicked and transferred to 96-well plates containing medium E (Stem cell technologies). Four days later hybridoma supernatants were transferred to 96-well plates and fresh medium E was added to the cells. To test the specificity of hybridoma supernatants for MHCII and p:MHCII, a decoy screening approach was employed. ELISA plates were coated with 50 ng well^−1^ either p63:IA^g7^ or InsB_10–23_:IA^g7^ (for p63:IA^g7^ antibodies), or 2W:IA^b^ or LLO:IA^b^, and then blocked with 1% BSA in PBS for 1 h. Hybridoma supernatants were mixed 1:1 with ELISA wash buffer (PBS+0.05% Tween20) and added to the p:MHCII-coated plates and incubated at 37 °C for 2 h. Media alone was used as a negative control while anti-IA^g7^ (clone 10–2.16 (ref. 28), BioXcell) or anti-IA^b^ (Y3P^29^, ATCC) were used as a positive control. For antibody detection the wells were incubated with HRP-conjugated goat anti-mouse IgG (BioLegend) diluted to 1:2,000 at room temperature for 2 h followed by addition of ABTS substrate solution (KPL) and detection by absorbance at 405 nm. Antibodies reacting to both p:MHCII monomers were considered specific for MHCII independent of peptide, while antibodies reacting with only p63:IA^g7^ or 2W:IA^b^ were considered p:MHCII specific.

### Antibody affinity measurements and sequencing

The affinity of the two novel MAb generated (FS1 and W6) were directly compared with known IA^g7^ or IA^b^ specific antibodies (10–2.16 (ref. 28) (BioXcell), Y3P^29^ (ATCC), Y-Ae^3^ (eBioscience) and AF6-120.1 (ref. 30) (BioLegend)) by Bio-Layer Interferometry by Precision Antibody (Columbia, MD). Antibody sequences were obtained using the SMARTer RACE cDNA Amplification Kit (Clontech) according to the manufacturer's instructions. Primers used for reverse transcription were GATTACGCCAAGCTTTATGCAAGGCTTACAACCACA (heavy chain), GATTACGCCAAGCTTCACAATTTTCTTGTCCACCTTGGTGC (heavy chain nested), GATTACGCCAAGCTTCTCATTCCTGTTGAAGCTCTTGACAAT (kappa light chain), GATTACGCCAAGCTTACACTCAGCACGGGACAAACTCTTCTC (lambda light chain 1, 4), GATTACGCCAAGCTTACACTCTGCAGGAGACAGACTCTTTTC (lambda 2,3).

### Proliferation and cytokine production

Cells were isolated from spleen and peripheral lymph nodes of NOD.BDC2.5 transgenic mice, the red blood cells were lysed by Tris-buffered ammonium chloride, and the cells were labelled with CFSE (ThermoFisher Scientific). Labelled cells were resuspended in complete DMEM media at a final concentration of 4 × 10^6 ^cells ml^−1^ and p63 or p31 peptide was added to a final concentration of 0.05 μM. Purified monoclonal antibody or 50 μl of hybridoma supernatant was added to each well containing 200 μl of cells in a 96-well plate, and incubated for 4 days at 37 °C with 5% CO_2_. Cells were collected, resuspended in 2.4G2 Fc block for 10 min at 4 °C, and stained at 1:100 dilution for 30 min at 4 °C with antibodies against CD4-BV510 (RM4-5, BD Biosciences), CD8α-BV650 (53–6.7, BD Biosciences), CD3e-PerCp-Cy5.5 (KT4, BD Biosciences), B220-ef450 (RA3-6B2, eBioscience), CD11c-ef450 (N418, eBioscience), CD11b-ef450 (M1/70, eBioscience), TCR Vβ4-PE (KT4, BD Biosciences) and dead cells were gated out using a viability ghost red dye (Tonbo), and run on a LSRII Fortessa X-20 flow cytometer (Becton Dickinson) and analyzed using FlowJo software (v10). For antibody dose–response curves, cells were isolated from NOD.BDC2.5 transgenic mice, labelled with CFSE as described above and cultured with p31 or p63 (0.05 μM) with varying doses of FS1 MAb or isotype control (BioXcell). After a 4-day incubation at 37 °C with 5% CO_2_, cells were collected for the flow cytometry, while supernatants were analyzed using ProcartaPlex Assay kit EPX170-26087-901 (eBioscience).

### Antibody staining of peptide-pulsed antigen-presenting cells

Purified monoclonal antibody was directly conjugated to Alexa Fluor-488 by protein labelling kit (ThermoFisher Scientific) according to the manufacturer's protocol. Splenocytes were isolated from NOD mice, red blood cells lysed and resuspended at in complete DMEM at 6 × 10^6 ^cells ml^−1^. p63 or OVA_141–160_ was added to the media at a final concentration of 40 μM and the cells were incubated for 1.5 h at 37 °C with 5% CO_2_. Cells were collected incubated in 2.4G2 for 10 min on ice and stained at 1:100 dilution for 30 min at 4 °C with FS1-AF488 and antibodies against CD4-BUV395 (GK1.5, BD Biosciences), CD8α-APC-ef780 (53–6.7, eBioscience), CD3ɛ-BV650 (145-2C11, BD Biosciences), B220-PE (RA3-6B, Tonbo), CD11c-PE-Cy (N418, eBioscience), CD11b-PerCp-Cy5.5 (M1/70, Tonbo), F4/80-APC (BM8.1, Tonbo), IA^g7^-biotin (10.2-16, BioXcell), SA-BV421 (BioLegend) and dead cells were gated out using Ghost violet 510 viability dye (Tonbo), and run on a LSRII Fortessa X-20 flow cytometer (Becton Dickinson) and analyzed using FlowJo software (v10).

### *In vivo* blockade of T-cell activation and tolerance

The NOD mice were administered 50 μg acetylated-p63 peptide with either 250 μg FS1 or Y-Ae MAb in PBS containing 2 μg LPS (Sigma) by i.v. injection in the tail vein. Four days post injection splenocytes were isolated and red blood cells lysed as above. Cells were stained with both APC and PE-conjugated p63:IA^g7^ tetramers followed by magnetic enrichment of double tetramer positive cells as previously described^14^,^37^. Enriched cells were incubated with 2.4G2 Fc block for 10 min at 4 °C and stained with surface antibodies at 1:100 dilution for 30 min at 4 °C against CD3e-APC-ef780 (17A2, eBioscience), CD4-BUV395 (GK1.5, BD Biosciences), CD8α-BV650 (53–6.7, BD Biosciences), CD44-BV786 (IM7, BD Biosciences), B220-PerCp-Cy5.5 (RA3-6B2, eBioscience), CD11b-PerCp-Cy5.5 (M1/70, Tonbo), CD11c-PerCp-Cy5.5 (N418, eBioscience) and Ghost violet 510 viability dye (Tonbo) for 30 min at 4 °C. The cells were then fixed using the Foxp3 staining buffer kit (eBioscience) and stained with anti-Ki67-PE-Cy7 (SolA15, eBioscience) antibodies for 1 h at 4 °C, and run on a LSRII Fortessa X-20 flow cytometer (Becton Dickinson) and analyzed using FlowJo software (v10). Pre-diabetic NOD mice received *in vitro* activated BDC2.5 T cells 19(ref. 19) followed by 30 million p63-cc intravenously and 500 micrograms of FS1 antibody on day 0 and 2 intravenously. Mice were monitored for diabetes by daily blood glucose measurements.

### Infections

C57BL/6 mice were injected intravenously with 10^7^ *actA*-deficient *Listeria monocytogenes* expressing 2W protein^25^, and on the same day injected intravenously with 500 μg of W6 (anti-2W:IA^b^) blocking antibody. Seven days later, splenocytes were isolated and stained as above and magnetically enriched for 2W:IA^b^ -PE tetramer. Wild type 129S1 mice were infected with *Salmonella* Typhimurium expressing 2W, and 14 days following infection mice were treated intravenously with 500μg of blocking W6 MAb. Thirty-five days post infection, the spleen was removed and made into a single cell suspension and 10% was plated for bacterial CFU with the addition of 0.1% Triton 27(ref. 27). The remaining cell suspension was used for enrichment of 2W specific cells as described above. Cells were run on a LSRII Fortessa X-20 flow cytometer (Becton Dickinson) and analyzed using FlowJo software (v10).

### Footpad immunization, antigen-presenting cell isolation and staining

The NOD hind limb footpads were injected with 100 μl total volume containing 200 μg of p63 or OVA_141–160_ peptide in PBS. One and half hours later the popliteal lymph nodes were removed, minced and digested in RPMI containing 2% FCS, collagenase D (40 U ml^−1^) and DNase I (250 μg ml^−1^) for 30 min at 4 °C. Cells were then washed with Hanks balanced salt solution containing 5 mM EDTA and 2% FCS, centrifuged and stained with surface antibodies and analyzed by flow cytometry as described above using the FS1-AF488 antibody.

### Ear pinna immunization, antigen-presenting cell isolation and staining

C57BL/6 mice were immunized intradermally in the ear pinna with 10 μg either Ovalbumin (OVA) or 2W-GFP plus 1 μg dmLT (a gift from J. Clements)^23^ or 10 μg CpG (Sigma). After 24 h, the cervical lymph nodes were removed, dissociated using a 100-μm mesh and mechanically disrupted, and digested with 300 Mandl U ml^−1^ Collagenase D (Roche Applied Sciences) for 30 min at 37 °C in 1 × PBS+2% FBS. Cells were then washed 1 × PBS+2% FBS, centrifuged and Fc receptors were blocked in 100 μl 2.4G2 hybridoma supernatant containing 2% rat and mouse serum for 10 min at room temperature. For surface staining, 1 μg of biotinylated W6 antibody was added to the cells and incubated on ice for 45 min, washed with PBS+2% FCS, followed by staining for 30 min at 4 °C with antibodies CD11c-PerCp-Cy5.5 (N418, BioLegend), CD11b-AF700 (M1/70, BioLegend), CD19-ef450 (1D3, eBioscience), MHCII-FITC (M5/114.15.2, BioLegend), DEC-205-PE (205yekta, eBioscience) and streptavidin-APC (BioLegend). All antibodies were used at 1:100 dilution except CD11c and CD19 at 1:40 and CD11b at 1:200. The cells were then run on a LSRII flow cytometer (Becton Dickinson) and analyzed using FlowJo software (v10).

### Statistical analysis

Data display and statistical analysis was conducted using Prism software (GraphPad Prism v6). Statistical significance was analyzed using the two-tailed Student's *t*-test for comparison of two mean. Values of *P*≤0.05 were considered significant.

### Data availability

Sequence data that support the findings of this study have been deposited in GenBank with the primary accession codes: FS1 heavy chain KU955585 (www.ncbi.nlm.nih.gov/nuccore/KU955585), FS1 light chain KU955586 (www.ncbi.nlm.nih.gov/nuccore/KU955586), W6 heavy chain KU955587 (www.ncbi.nlm.nih.gov/nuccore/KU955587) and W6 light chain KU955588 (www.ncbi.nlm.nih.gov/nuccore/KU955588).

## Additional information

**How to cite this article**: Spanier, J. A. *et al.* Efficient generation of monoclonal antibodies against peptide in the context of MHCII using magnetic enrichment. *Nat. Commun.* 7:11804 doi: 10.1038/ncomms11804 (2016).

## Figures and Tables

**Table 1:** Efficiency of generating hybridomas producing p:MHCII-specific antibodies.

**Antigen**	**# Mice immunized**	**# Hybridomas screened**	# Hybridomas^+^for MHCII (%)*	**# Hybridomas^+^for peptide:MHCII (%)**
p63:IA^g7^	5	190	32 (16.8)	11 (5.8)†
2W:IA^b^	2	576	234 (40.6)	5 (0.9)†
LLO:IA^b^	2	576	236 (40.9)	32 (13.5)*
Ins:IA^g7^	2	576	22 (3.8)	11 (1.9)*
p31:IA^g7^	2	480	21 (4.4)	14 (2.9)*
mimetope:IA^g7^	2	480	21 (4.4)	12 (2.5)*

^*^Specificity as defined by positive ELISA plate compared to decoy pMHCII complex.

^†^Specificity as defined by functional assay blocking T-cell proliferation or MAb binding to APCs.

**Table 2:** Comparison of anti-p:MHCII antibody affinities.

**Antibody clone**	**Antigen**	***K***_**D**_**(M)**
FS1	p63:IA^g7^	1.7 × 10^−11^
10–2.16 (ref. 28)	p63:IA^g7^	2.9 × 10^−9^
W6	2W:IA^b^	3.4 × 10^−9^
AF6-120.1 (ref. 30)	2W:IA^b^	7.1 × 10^−9^
Y3P (ref. 29)	2W:IA^b^	1.9 × 10^−9^
Y-Ae (ref. 3)	Eα_52–68_:IA^b^	4.8 × 10^−10^

## References

[b1] Kohler G. & Milstein C. Continuous cultures of fused cells secreting antibody of predefined specificity. Nature 256, 495–497 (1975). 1172191 10.1038/256495a0

[b2] Yokoyama W. M. *et al.* Production of monoclonal antibodies. Curr. Protoc. Immunol. 102, Unit 2.5 (2013). 24510488 10.1002/0471142735.im0205s102

[b3] Murphy D. B. *et al.* A novel MHC class II epitope expressed in thymic medulla but not cortex. Nature 338, 765–768 (1989). 2469959 10.1038/338765a0

[b4] Zhang L. *et al.* Monoclonal antibody blocking the recognition of an insulin peptide–MHC complex modulates type 1 diabetes. Proc. Natl Acad. Sci. USA 111, 2656–2661 (2014). 24550292 10.1073/pnas.1323436111 PMC3932899

[b5] Wolpl A. *et al.* Human monoclonal antibody with T-cell-like specificity recognizes MHC class I self-peptide presented by HLA-DR1 on activated cells. Tissue Antigens 51, 258–269 (1998). 9550326 10.1111/j.1399-0039.1998.tb03100.x

[b6] Muraille E. *et al.* Direct visualization of peptide/MHC complexes at the surface and in the intracellular compartments of cells infected in vivo by *Leishmania* *major*. PLoS Pathog. 6, e1001154 (2010). 20976202 10.1371/journal.ppat.1001154 PMC2954901

[b7] Baldwin K. K., Reay P. A., Wu L., Farr A. & Davis M. M. A T-cell receptor-specific blockade of positive selection. J. Exp. Med. 189, 13–24 (1999). 9874560 10.1084/jem.189.1.13 PMC1887687

[b8] Zhong G., Reis e Sousa C. & Germain R. N. Production, specificity, and functionality of monoclonal antibodies to specific peptide-major histocompatibility complex class II complexes formed by processing of exogenous protein. Proc. Natl Acad. Sci. USA 94, 13856–13861 (1997). 9391117 10.1073/pnas.94.25.13856 PMC28397

[b9] Dadaglio G., Nelson C. A., Deck M. B., Petzold S. J. & Unanue E. R. Characterization and quantitation of peptide–MHC complexes produced from hen egg lysozyme using a monoclonal antibody. Immunity 6, 727–738 (1997). 9208845 10.1016/s1074-7613(00)80448-3

[b10] Pape K. A., Taylor J. J., Maul R. W., Gearhart P. J. & Jenkins M. K. Different B cell populations mediate early and late memory during an endogenous immune response. Science 331, 1203–1207 (2011). 21310965 10.1126/science.1201730 PMC3993090

[b11] Newman J., Rice J. S., Wang C., Harris S. L. & Diamond B. Identification of an antigen-specific B cell population. J. Immunol. Methods 272, 177–187 (2003). 12505722 10.1016/s0022-1759(02)00499-4

[b12] Moon J. J. *et al.* Tracking epitope-specific T cells. Nat. Protoc. 4, 565–581 (2009). 19373228 10.1038/nprot.2009.9 PMC3517879

[b13] Moon J. J. *et al.* Naive CD4(+) T cell frequency varies for different epitopes and predicts repertoire diversity and response magnitude. Immunity 27, 203–213 (2007). 17707129 10.1016/j.immuni.2007.07.007 PMC2200089

[b14] Pauken K. E. *et al.* Cutting edge: type 1 diabetes occurs despite robust anergy among endogenous insulin-specific CD4 T cells in NOD mice. J. Immunol. 191, 4913–4917 (2013). 24123682 10.4049/jimmunol.1301927 PMC3987747

[b15] Fife B. T. & Bluestone J. A. Control of peripheral T-cell tolerance and autoimmunity via the CTLA-4 and PD-1 pathways. Immunol. Rev. 224, 166–182 (2008). 18759926 10.1111/j.1600-065X.2008.00662.x

[b16] Pauken K. E., Jenkins M. K., Azuma M. & Fife B. T. PD-1, but not PD-L1, expressed by islet-reactive CD4+ T cells suppresses infiltration of the pancreas during type 1 diabetes. Diabetes 62, 2859–2869 (2013). 23545706 10.2337/db12-1475 PMC3717847

[b17] Haskins K., Portas M., Bradley B., Wegmann D. & Lafferty K. T-lymphocyte clone specific for pancreatic islet antigen. Diabetes 37, 1444–1448 (1988). 2458291 10.2337/diab.37.10.1444

[b18] Katz J. D., Wang B., Haskins K., Benoist C. & Mathis D. Following a diabetogenic T cell from genesis through pathogenesis. Cell 74, 1089–1100 (1993). 8402882 10.1016/0092-8674(93)90730-e

[b19] Fife B. T. *et al.* Interactions between PD-1 and PD-L1 promote tolerance by blocking the TCR-induced stop signal. Nat. Immunol. 10, 1185–1192 (2009). 19783989 10.1038/ni.1790 PMC2778301

[b20] Taylor J. J. *et al.* Deletion and anergy of polyclonal B cells specific for ubiquitous membrane-bound self-antigen. J. Exp. Med. 209, 2065–2077 (2012). 23071255 10.1084/jem.20112272 PMC3478923

[b21] Judkowski V. *et al.* Identification of MHC class II-restricted peptide ligands, including a glutamic acid decarboxylase 65 sequence, that stimulate diabetogenic T cells from transgenic BDC2.5 nonobese diabetic mice. J. Immunol. 166, 908–917 (2001). 11145667 10.4049/jimmunol.166.2.908

[b22] Rees W. *et al.* An inverse relationship between T cell receptor affinity and antigen dose during CD4(+) T cell responses in vivo and in vitro. Proc. Natl Acad. Sci. USA 96, 9781–9786 (1999). 10449771 10.1073/pnas.96.17.9781 PMC22287

[b23] Norton E. B. *et al.* The novel adjuvant dmLT promotes dose sparing, mucosal immunity and longevity of antibody responses to the inactivated polio vaccine in a murine model. Vaccine 33, 1909–1915 (2015). 25765967 10.1016/j.vaccine.2015.02.069

[b24] Fife B. T. *et al.* Insulin-induced remission in new-onset NOD mice is maintained by the PD-1-PD-L1 pathway. J. Exp. Med. 203, 2737–2747 (2006). 17116737 10.1084/jem.20061577 PMC2118162

[b25] Ertelt J. M. *et al.* Selective priming and expansion of antigen-specific Foxp3-CD4+ T cells during *Listeria monocytogenes* infection. J. Immunol. 182, 3032–3038 (2009). 19234199 10.4049/jimmunol.0803402 PMC2677098

[b26] Uzzau S., Figueroa-Bossi N., Rubino S. & Bossi L. Epitope tagging of chromosomal genes in *Salmonella*. Proc. Natl Acad. Sci. USA 98, 15264–15269 (2001). 11742086 10.1073/pnas.261348198 PMC65018

[b27] Nelson R. W., McLachlan J. B., Kurtz J. R. & Jenkins M. K. CD4+ T cell persistence and function after infection are maintained by low-level peptide:MHC class II presentation. J. Immunol. 190, 2828–2834 (2013). 23382562 10.4049/jimmunol.1202183 PMC3594488

[b28] Oi V. T., Jones P. P., Goding J. W., Herzenberg L. A. & Herzenberg L. A. Properties of monoclonal antibodies to mouse Ig allotypes, H-2, and Ia antigens. Curr. Top. Microbiol. Immunol. 81, 115–120 (1978). 567555 10.1007/978-3-642-67448-8_18

[b29] Janeway C. A. Jr. *et al.* Monoclonal antibodies specific for Ia glycoproteins raised by immunization with activated T cells: possible role of T cellbound Ia antigens as targets of immunoregulatory T cells. J. Immunol. 132, 662–667 (1984). 6228596

[b30] Lorber M. I., Loken M. R., Stall A. M. & Fitch F. W. I-A antigens on cloned alloreactive murine T lymphocytes are acquired passively. J. Immunol. 128, 2798–2803 (1982). 6804568

[b31] Rudensky A., Rath S., Preston-Hurlburt P., Murphy D. B. & Janeway C. A. Jr. On the complexity of self. Nature 353, 660–662 (1991). 1656278 10.1038/353660a0

[b32] Murphy D. B. *et al.* Monoclonal antibody detection of a major self peptide. MHC class II complex. J. Immunol. 148, 3483–3491 (1992). 1375245

[b33] Pauken K. E. *et al.* Cutting edge: identification of autoreactive CD4+ and CD8+ T cell subsets resistant to PD-1 pathway blockade. J. Immunol. 194, 3551–3555 (2015). 25769925 10.4049/jimmunol.1402262 PMC4390507

[b34] Lee S. J. *et al.* Temporal expression of bacterial proteins instructs host CD4 T cell expansion and Th17 development. PLoS Pathog. 8, e1002499 (2012). 22275869 10.1371/journal.ppat.1002499 PMC3262010

[b35] Nelson R. W. *et al.* T cell receptor cross-reactivity between similar foreign and self peptides influences naive cell population size and autoimmunity. Immunity 42, 95–107 (2015). 25601203 10.1016/j.immuni.2014.12.022 PMC4355167

[b36] Day C. L. *et al.* Ex vivo analysis of human memory CD4 T cells specific for hepatitis C virus using MHC class II tetramers. J. Clin. Invest. 112, 831–842 (2003). 12975468 10.1172/JCI18509 PMC193667

[b37] Tubo N. J. *et al.* Single naive CD4(+) T cells from a diverse repertoire produce different effector cell types during infection. Cell 153, 785–796 (2013). 23663778 10.1016/j.cell.2013.04.007 PMC3766899

